# *Acmella oleracea* and* Achyrocline satureioides* as Sources of Natural Products in Topical Wound Care

**DOI:** 10.1155/2016/3606820

**Published:** 2016-09-29

**Authors:** Lais Thiemi Yamane, Eneida de Paula, Michelle Pedroza Jorge, Verônica Santana de Freitas-Blanco, Ílio Montanari Junior, Glyn Mara Figueira, Luís Adriano Anholeto, Patricia Rosa de Oliveira, Rodney Alexandre Ferreira Rodrigues

**Affiliations:** ^1^Chemical, Biological and Agricultural Research Center (CPQBA), University of Campinas, Campinas, SP, Brazil; ^2^Biology Institute, Department of Biochemistry and Tissue Biology, University of Campinas, Campinas, SP, Brazil; ^3^Institute of Biosciences, Department of Biology, Sao Paulo State University, Rio Claro, SP, Brazil

## Abstract

The Brazilian forests have one of the world's biggest biodiversities.* Achyrocline satureioides* (macela) and* Acmella oleracea* (jambu) are native species from Brazil with a huge therapeutic potential, with proved anti-inflammatory and anesthetic action, respectively. The jambu's crude extract after depigmentation with activated charcoal and macela's essential oil were incorporated in a film made with hydroxyethyl cellulose. Those films were evaluated by mechanical test using a texturometer and anti-inflammatory and anesthetic activities by* in vivo* tests: wound healing and antinociceptive. The film containing the highest concentration of depigmented jambu's extract and macela's essential oil obtained an anesthesia time of 83.6 (±28.5) min longer when compared with the positive control EMLA®; the same occurred with the wound healing test; the film containing the highest concentration had a higher wound contraction (62.0% ± 12.1) compared to the positive control allantoin and the histopathological analysis demonstrated that it increases collagen synthesis and epidermal thickening. The results demonstrate that the films have a potential use in skin wounds, pressure sore, and infected surgical wounds treatment.

## 1. Introduction

The wound dressing market has grown significantly over last 10 years; however fewer dressings have all the required properties for the purpose of wound protection and healing. Natural polymers are of special interest due to low toxicity, high absorption, and swelling capacities; moreover, various active compounds can be incorporated into these polymers [1].

The hydroxyethyl cellulose (HEC) is considered the most abundant natural polymer, renewable, soluble in water, with low toxicity, film-forming, with good stability from pH 2 and pH 12, is relatively inexpensive, and is widely used in various types of dressings to have hemostatic property [2–4]. The prevalence of chronic wounds in the general population is relatively high; improving wound healing, severe burns, and postoperative remains a challenge due to the high cost of existing therapies [5]. The use of opioids is widely used as anesthetics for postoperative and deep wounds, but there are disadvantages as possible side effects (respiratory depression, nausea, vomiting, constipation, and urinary retention) [6] and antibiotics are generally used to prevent and treat infections; nonetheless their activity is gradually diminishing due to the rise of resistance to these drugs [1].

Topical formulations are noninvasive and with few side effects; a good example is patches, a topical adhesive that have constant and prolonged release, avoiding peak plasma levels, and may reduce the duration of pain [7]. The patch with plant extracts has been used to reduce inflammation and aid in healing [5].

Brazilian biome contains more than 20% of the biodiversity of world [8], with a wide variety of available molecules for human therapy in pharmaceutical, food, cosmetic, and veterinary fields [9]. Two species that are used in popular culture and native of Brazil and South America were used for this study,* Achyrocline satureioides* Lam. (DC) (*Asteraceae*) known as macela or marcela, used in folk medicine as an analgesic, sedative, and abdominal cramping. There is scientific evidence of anti-inflammatory, antispasmodic, and antioxidant action [10]. The essential oil compounds have been identified as *α*-pinene, copaene, and the isomers *β*-caryophyllene and *α*-caryophyllene (or *α*-humulene); the last two are the major compounds present in essential oil of plants in Brazil [11]. Its main component of interest is the *α*-humulene, responsible for anti-inflammatory activity [12].* Acmella oleracea* (L.) RK Jansen (*Asteraceae*), popularly known in Brazil as jambu, is widely used in cooking and also popularly used to treat toothaches, tuberculosis, and anemia and as appetite stimulant [13]; these activities are attributed to bioactive compounds, coumarins (scopoletin), triterpenoids, essential oils (limonene and *β*-caryophyllene) amino acids, phytosterols, phenolic compounds (vanillic acid, trans-ferulic acid, and trans-isoferulic acid), and N-alkylamides; its main component is spilanthol responsible for anesthetic activity [14–17].

The use of an anesthetic and anti-inflammatory is important to reduce pain and have good wound healing, for reducing the risk of infection and further complications. It is also reported that inflammatory mediators such as histamine and prostaglandin E2 may be related to the inhibition of collagen synthesis and fibroblast proliferation, important components for wound healing [18, 19].

The aim of this work was to develop and to evaluate an anesthetic and anti-inflammatory bioadhesive film containing jambu extract and macela essential oil, by studying its mechanical properties,* in vitro* skin permeation characteristics, and* in vivo* performance, in comparison with commercial formulations.

## 2. Materials and Methods

### 2.1. Chemicals

HEC and *α*-humulene standard (96% purity) were purchased from Sigma-Aldrich Co., Saint Louis, USA. Spilanthol standard (88.5%) purity was purchased from Chromadex, California, USA. Glycerin was purchased from Vetec Química Fina, Rio de Janeiro, Brazil. Transcutol® (ethoxydiglycol) was purchased from Gattefossé®, Lyon, France. Activated carbon was purchased from Carbomafra®, Curitiba, Brazil. Diatomaceous Earth and Tween 80® were purchased from Synth, São Paulo, Brazil. Allantoin was purchased from Fagron, Hong Kong, China.

All other reagents were of analytical grade.

### 2.2. Plant Material

The plant material was sown and grown in the experimental field of the Multidisciplinary Center for Chemical, Biological and Agricultural Research (CPQBA)/UNICAMP, located in the city of Paulinia, Brazil. This project was authorized by the Council of the Genetic Heritage Management, under protocol number 01232/2014-1.

### 2.3. Production of Jambu Ethanolic Extract and Treatment with Activated Charcoal

Jambu aerial parts were collected, dried, milled, and extracted 3 times with 96° GL ethanol (1 : 5 w/v) for 1.5 h. The three fractions were pooled and filtered in Buchner funnel with filter paper under vacuum. Then the crude extract was weighed and treated with 4% of activated charcoal; the mixture crude extract and activated charcoal were placed in a warm bath at 40°C with constant stirring for one hour. The extract was filtered with diatomaceous earth (Celite®) and evaporated at 40°C under reduced pressure (model R-215, Büchi) and then lyophilized until constant weight. Solvent-free extract was stored in a freezer until use.

### 2.4. Macela Volatile Oil Production

The volatile oil was obtained from fresh inflorescences in Clevenger apparatus by hydrodistillation held for a period of 2 h according González and Marioli [20]. The oil was separated from the water in a separation funnel and anhydrous sodium sulfate was added to remove residual water. The oil was stored in a freezer until use.

### 2.5. GC-MS Analysis

The analytical monitoring of spilanthol and *α*-humulene in the extract, essential oil, and films was performed by gas chromatograph coupled with a mass detector (CG-EM, Hewlett Packard 5890, series II, mass selective detector 5970 EI 70 eV) equipped with a fused silica column WCOT, HP5-MS, Agilent®, with 30 m × 0.25 mm × 0.25 *μ*m of dimension. The analysis conditions were injection temperature: 220°C, detector temperature: 250°C, and temperature program: 60–240°C (3°C/min). The identification and quantification of spilanthol and *α*-humulene were made by the external standard method with commercial analytical standard [21].

### 2.6. Preparation and Characterization of HEC Films

To produce the films, 2.5% of HEC was added in 100 mL ultrapure water and the resulting mixture kept under magnetic stirring and heating at 55°C until complete dissolution. The mixture rested until it reaches room temperature and then jambu depigmented crude extract (10.0 and 15.0%), macela's essential oil (1.0 and 1.5%), 5.0% of Transcutol, 0,7% of Tween 80, and 0.7% of glycerin were added. The homogeneous solution was transferred to Nylon plates, which were dried in an oven at 40°C for 24 h.

After drying, the films were removed from the plates and cut with a punch with 17 mm in diameter, size compatible with the wound healing test and antinociceptive activity tests. The films were weighed on an analytical scale (Mettler Toledo, São Paulo, Brazil) and their thickness was measured with a digital caliper (model Cal II, Tesa, Renens, Switzerland).

### 2.7. Mechanical Resistance Properties

Mechanical properties of the films with extract and essential oil and placebo film (HEC, Transcutol, Tween 80, and glycerin) were evaluated using a texturometer from Stabile Micro Systems (model TA-TX Enhanced, Godalming, United Kingdom) evaluating resistance to perforation, relaxation, and resilience. The determination parameters were set as distance of 30 mm for the perforation resistance tests, distance of 2 mm for both resilience and relaxation tests, and a force of 5 g for all tests. All determinations were performed in triplicate [22].

### 2.8. Pharmacological Tests

#### 2.8.1. Animals

Male Wistar rats (250–300 g) and male Swiss mice (25–40 g) were obtained from CEMIB-UNICAMP (Multidisciplinary Center for Biological Research, State University of Campinas, UNICAMP, Campinas, São Paulo, Brazil). All animals were housed in polycarbonate cages, under a climate-controlled environment (22 ± 3°C and relative humidity 30–70%), a light/dark 12 h cycle, and feed* ad libitum* with convectional standard palletized laboratory ration (Nuvilab®) and water. Protocols were approved by the UNICAMP Institutional Animal Care and Use Committee, which follows the recommendations of the Guide for the Care and use of Laboratory Animals (CEUA number 3342-1).

#### 2.8.2. Evaluation of Wound Healing Activity in Rats

This assay was performed according to Jorge et al. [23]. Wistar rats were divided in 6 groups of 5 animals each: group I, saline 0.9% (0.5 mL/animal, negative control); group II, allantoin (100 mg/animal, positive control); group III, FI: film with 10.0% of depigmented jambu's extract with 1% of macela's essential oil (250 mg/animal); group IV, FII: film with 15.0% of depigmented jambu's extract with 1,5% of macela's essential oil (250 mg/animal); group V, FIII: placebo film. Cutaneous ulcers were visually examined daily; also photos were taken and the percentage of reduction of the initial lesion area were calculated, by ImageJ® program.

At the end of the experiment the animals were sacrificed by deepening anesthesia (thiopental) and skin samples were removed (epidermis, dermis and hypodermis) of the same treatment site. These were removed from groups 4, allantoin, FI, FII, and FIII, placed in 10% formalin solution and stained with Trichrome Masson and Hematoxylin/Eosin. By storage issues the saline group was lost. The stained slides were examined by light microscopy.

#### 2.8.3. Tail-Flick Test in Mice

This assay was performed according to de Araujo et al. [24], with some modifications, such as cut-off time, intervals measurement, and animal employed. The animals were placed in a horizontal acrylic restraint with the distal portion of the tail free and exposed to heat from a lamp (55 ± 1°C). The timer stopped when the exposed rat tail flicks, and the interval between switching on the light and flick of the tail was recorded (latency time). A 10 s cut-off time was used to avoid thermal injury, and the baseline (normal response to the noxious stimulus) was recorded before starting the experiments. The animals were divided into 4 groups: group I, FI; group II, FII; group III, FIII; and group IV, EMLA 150 mg/animal (positive control). The films and EMLA were applied to 2 cm from the base of the animal's tail for 5 min; after its removal, the tail was exposed to light focused on the same region where the film was applied and the test was started after the tail removal. The measurements were made every 15 min until the animal returned to its baseline response to noxious stimuli.

### 2.9. Statistical Analysis

Characterization and* in vivo* pharmacological data were expressed as percentage or mean ± SD and analyzed by one-way analysis of variance (one-way ANOVA) with Tukey-Kramer post hoc tests using Graph Pad Instat (Graph Pad Software Inc., USA).

## 3. Results

### 3.1. Yield from Extraction Processes and GC-MS Analysis

The average yield of depigmented jambu crude extract on a dry basis was 3.7% and average yield of macela's essential oil was 0.04%.

**Figure 1:**
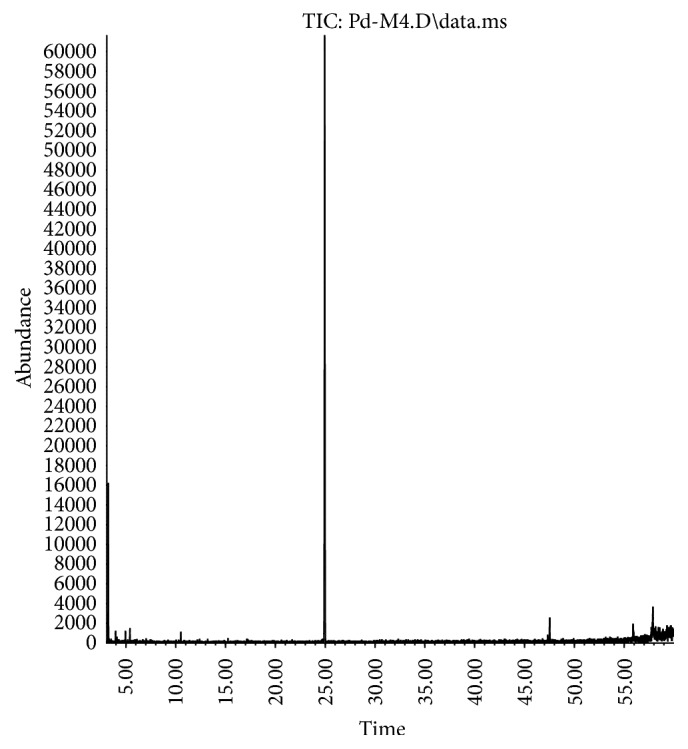
GC-MS chromatogram of commercial analytical standard of *α*-humulene.

**Figure 2:**
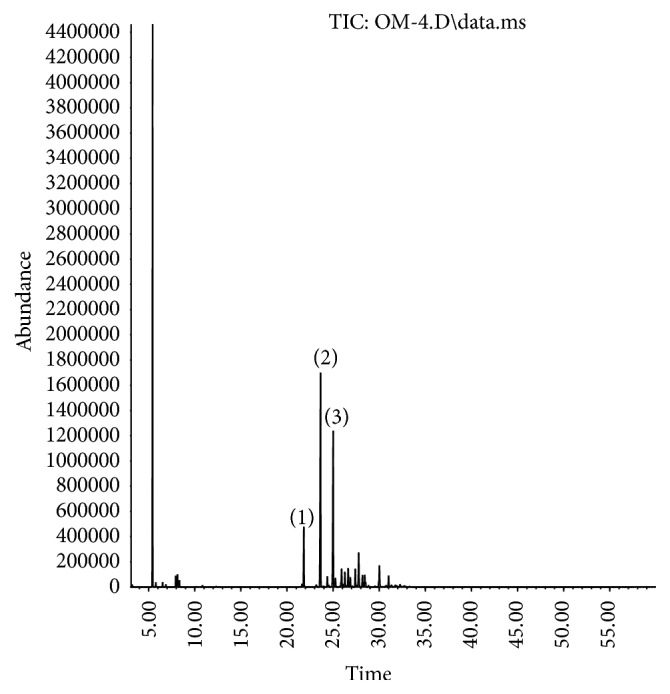
GC-MS chromatogram of macela's essential oil. Major compounds are spotlighted by (1) copaene, (2) *β*-caryophyllene, and (3) *α*-humulene.

**Figure 3:**
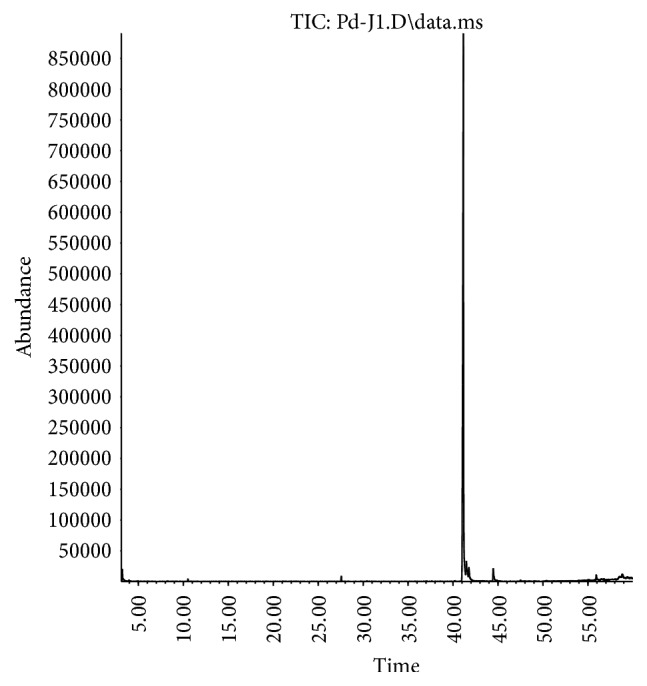
GC-MS chromatogram of commercial analytical standard of spilanthol.

**Figure 4:**
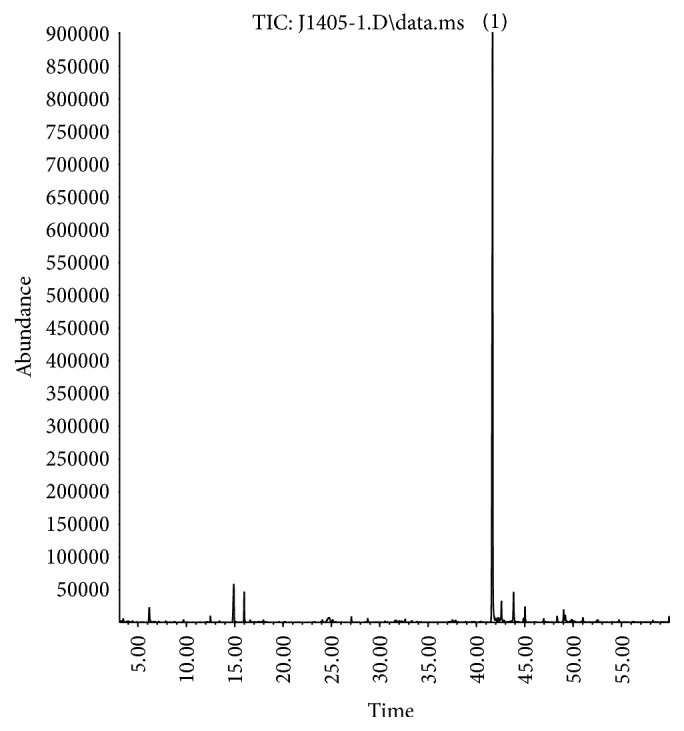
GC-MS chromatogram of jambu extract. Spilanthol peak is spotlighted by (1).

The chromatograms obtained by GC-MS analysis of commercial analytical standards are illustrated in Figure 1 (*α*-humulene) and Figure 3 (spilanthol) and the macela's essential oil and jambu extract in Figures 2 and 4, respectively.

### 3.2. Preparation and Characterization of HEC Films

The formulations remained homogeneous, dried after 24 hours in an oven, and had a good appearance. All films were characterized by weight and thickness; the weight of the film with the highest concentration was in the range of 0.2506 ± 0.0127 g/film and the film with lower concentration was in the range of 0.2509 ± 0.0135 g/film and the HEC film was in the range of 0.1972 ± 0.0134 g/film and the thickness was in the range of 0.9308 ± 0.083 mm for the film with the highest concentration and 0.9292 ± 0.125 mm for the film with lower concentration and 0.7901 ± 0.058 mm for the HEC film.

### 3.3. Mechanical Resistance Properties

The results produced during evaluation of the mechanical resistance of the films with respect to perforation, relaxation, and resilience are displayed in Table 1. The FIII exhibited a higher resistance than the other two formulations containing extract and essential oil. There was no significant difference statistical between treatments FI and FII.

### 3.4. Evaluation of Wound Healing Activity in Rats

**Figure 5:**
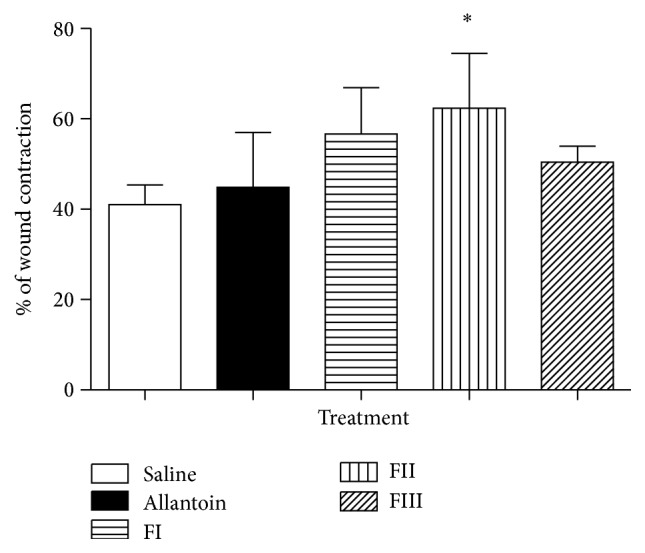
Evaluation of wound contraction in different treatments during 10 days (^*∗*^ *p* > 0.05, *n* = 5).

Macroscopic evaluation of the wounds was performed on days 0 to 10. After 10 days of treatment, the animals treated with saline were in pain and a purulent exudate was found with reddish color, while the other groups had a more docile behavior and also there was the formation of crusts without pus. Wound healing of the skin incision was determined by the percentage of wound surface covered by regenerating epidermis and is illustrated in Figure 5. The treatment FII had the highest percentage of wound contraction 62.38% ± 12.11, with statistical significance with saline. Treatments FI (56.65% ± 10.23) and FIII (47.20% ± 4.3) had a higher percentage of wound contraction than the control (Allantoin: 44.83 ± 4.32). Treatment with saline, as expected, showed the lowest percentage of contraction (41.00% ± 4.3). The control and the formulations showed a progressive reduction in wounded area during the experiment.

Histopathological analysis was made in an optical microscope (Leica DM 750, Illinois, USA) coupled to a digital camera (Leica ICC50HD, Illinois, USA).

**Figure 6:**
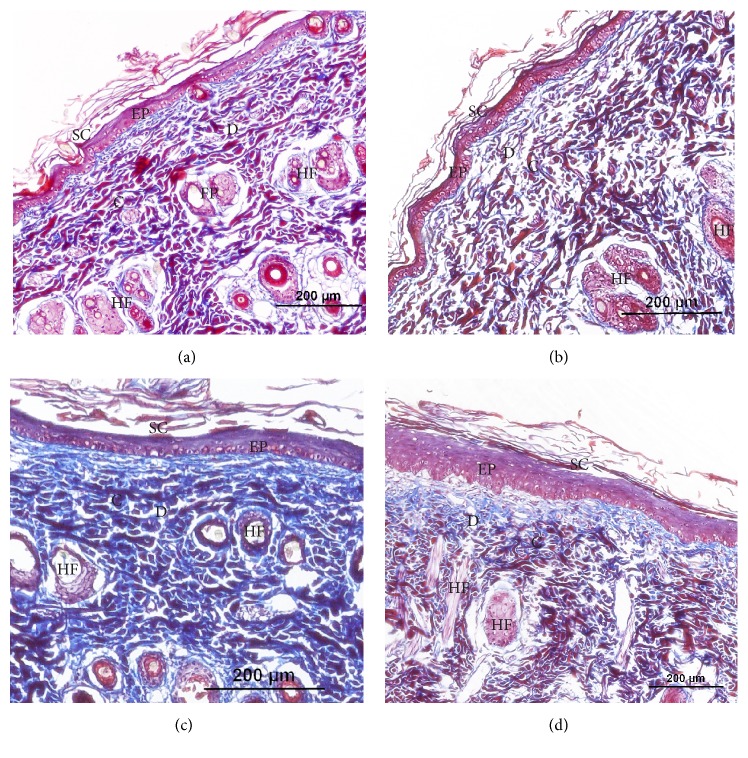
Histological sections from the wound area stained with Trichrome Masson. Slides were observed by optical microscopy at 200 *μ*m. (a) Allantoin-positive control; (b) placebo film-negative control or FIII film; (c) FI film; (d) FII film. SC: stratum corneum; EP: epidermis; D: dermis; C: collagen; HF: hair follicle.

**Figure 7:**
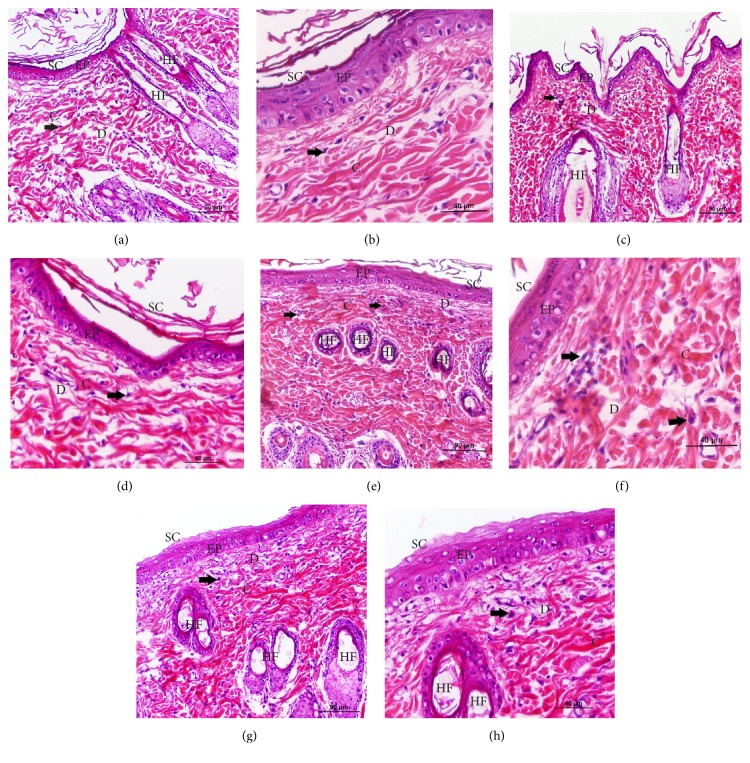
Histological sections from the wound area stained with Hematoxylin/Eosin. Slides were observed by optical microscopy at two different magnifications 90 and 40 *μ*m. (a/b): allantoin-positive control; (c/d): placebo film-negative control or FIII film; (e/f): FI film; (g/h): FII film. SC: stratum corneum; EP: epidermis; D: dermis; C: collagen; HF: hair follicle and the arrow indicate the fibroblast's core.

The results from the* in vivo* study according to the images demonstrated that the positive control (Figures 6(a), 7(a), and 7(b)) and treatment FIII (Figures 6(b), 7(c), and 7(d)) did not help with the improvement of fibroblast production and the rearrangement of collagen fibers, when compared with treatments FI/FII. The treatments FI (Figures 6(c), 7(e), and 7(f)) and FII (Figures 6(d), 7(g), and 7(h)) showed a better distribution of collagen fibers (C), demonstrated by the purplish blue color sections stained with Trichrome Masson (TM) and by the intense pink color stained with Hematoxylin/Eosin (HE). Furthermore, both these treatments showed higher fibroblasts production, demonstrated by their cores as indicated by arrows in Figure 7. Regarding the thickening of the epidermis (EP), the placebo film showed the smallest epidermis thickness, whereas the other treatments showed a greater thickening of this region.

### 3.5. Tail-Flick Test

**Figure 8:**
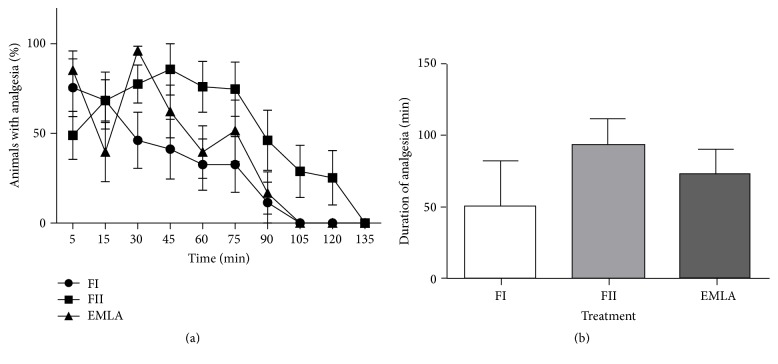
Percent of animals with analgesia* versus* time (a) and duration of analgesia (b). FI: film with 10.0% of jambu extract and 1.0% of macela essential oil; FII: film with 15.0% of jambu extract and 1,5% of macela essential oil. There was no statistical significance between the groups.

Figure 8 illustrates the analgesic efficacy of the EMLA commercial cream and the two different films (*p* > 0.05, *n* = 6). EMLA had a higher percentage of animals with analgesia but FII obtained duration of analgesia higher than the control. FII had the lowest duration and animals with analgesia. As expected, no signs of analgesic effects were observed in the animals treated with placebo films (data not shown).

## 4. Discussion

The activated charcoal is widely used in industrial processes for its ability to absorb a large number of substances, such as organic compounds, heavy metals, and colored substances, and be biocompatible and nontoxic [25]. The jambu's depigmented extract yield is not reported in the literature. Studies made of our research group showed that the removal of compounds in the ethanolic extract of jambu, in most the chlorophyll, increases espilantol percentage in relation to the crude extract jambu even with reduced mass of the extract, since most of the components retained in the charcoal were pigments. Other authors have reported the effectiveness of activated charcoal in the removal of pigments, but none of jambu [21].

Macela's essential oil yield was below average described in literature, from 0.4 to 0.2%. This little yield could be justified by the harvesting period of the plant and type of soil used. It is known that the yield also varies according to the crop region [11].

Analysis by GC-MS showed that the compounds of interest, *α*-humulene and spilanthol, were present in jambu's extract and macela's essential oil, respectively, and also in the films.

The extract and the essential oil were incorporated into a film because they have some advantages over conventional dosage forms to avoid hepatic first-pass metabolism, improving the drug bioavailability and decreasing the dose frequency [26]. We used adjuvants to improve the properties of the films and to facilitate the solubilization of the extract and essential oil. Glycerin was used as a plasticizer; Tween 80 as emulsifier, and Transcutol as solubilizing/facilitating the penetration of active skin [27–29]. The polymer used was the HEC, by the aforementioned properties, for being compatible with the adjuvants, jambu's extract and macela's essential oil; it remained homogeneous after drying. Other polymers were tested in different concentrations, such as chitosan and polyvinyl alcohol. Also other ingredients like adjuvants were exhausted tested, but none of them remained homogeneous (data not shown).

To determine the physical properties of the films these were evaluated with respect to perforation, relaxation, and resilience. The mechanical properties of the films are mainly related with the polymer's ability to form bonds in polymer chains, making their separation difficult when subject to mechanical forces. The placebo film (FIII film) exhibited a higher resistance than the films with extract and essential oil; it presented higher values relative to perforation, resilience, and relaxation, as expected since there is no addition of extract and oil; consequently the film is more rigid. Hence, it may be concluded that the presence of jambu's extract and macela's essential oil improved their viscoelastic characteristics of deformation and molecular relaxation. Compared with another study of polyvinyl alcohol films, the films containing extract and essential oil are more malleable and easiest to cover the wound [20].

Wound healing is the process of repair following injury to the skin and other soft tissues. It is a complex and dynamic process of restoring cellular structures and tissue layers in damaged tissue as closely as possible to its normal and original state [30]. The formulations containing jambu's extract and macela's essential oil improved wound contraction and closure by the absorptive capacity of fluids. The formulation FII had the highest percentage of wound contraction and formulation FIII was better than the control, proving that the HEC is a great polymer for wound healing. Spilanthol and *α*-humulene already have anti-inflammatory action proven [31, 32] but their healing activity has not been studied. The histopathological analysis showed that, in just 10 days of treatment, the treatments with the FI and FII films were beneficial for the regeneration and reorganization of structures of the skin when compared with placebo film and allantoin; this is confirmed by the regular arrangement of collagen fibers and thickening epidermis in Figures 6 and 7. This result suggests that the jambu's extract and macela's essential oil helped in collagen deposition and decrease the time of the healing process. Moreover, it was not possible to find any evidence of tissue damage, inflammation, or fibrosis in the rat skin.

In a study with another species but still natural product, the authors employed* Hibiscus rosa *extract in an ointment base and demonstrated that in 10 days the wound contracted about 75.0%, while the positive control, nitrofurazone ointment, contracted on average 45.0% [33]. One study from Asian medicine with ethanol extract and essential oil of* Murraya koenigii* proved that the administered essential oil helped with the wound contraction at 36.0%, the treatment with ethanol extract at 67.0%, and the ointment standard used at 37.0%, at the tenth day of treatment, but it was possible to observe the presence of collagen in the histological slides only on the 14th day [34]. These results demonstrate that the use of essential oils and extracts is important in wound healing; therapeutic and wound healing test corroborates this statement.

The antinociceptive activity was dose-dependent with only 5 min of application; formulation FII had a longer time of anesthesia than the EMLA cream, but formulation FI obtained a shorter time than EMLA The topical EMLA cream was chosen as a positive control because it is the topical anesthetic most used clinically and has an average duration of anesthesia 90 min after application [35].

Moreover, the antinociceptive activity of jambu's extract is due to the presence of spilanthol, an alkylamine in a high percentage in the extract. Other authors demonstrate the efficacy of jambu in antinociceptive test intraperitoneally [36]; this demonstrates the safety of jambu's extract.

## 5. Conclusion

The FII film with 15.0% of jambu extract treated with activated charcoal and 1.5% macela's essential oil had the best results. The mechanical resistance tests displayed that films with extract and essential oil are more malleable, making it easier to cover wounds when compared with other films, indicating that the films may be utilized in different locations of the human body with skin lesions. It had better healing activity and collagen deposition compared with the positive control, allantoin. It also had the best result in the antinociceptive activity test compared with EMLA, a topical anesthetic most commonly used clinically. These results demonstrate the great potential of films as wound dressing.

## Figures and Tables

**Table 1:** Results from the mechanical resistance tests (kg) performed to films, relative to perforation, relaxation, and resilience, as a function of applied force (kg) (AUC).

Films	Perforation		Relaxation		Resilience	
	Force (g)	AUC (g/s)	Force (g)	AUC (g/s)	Force (g)	AUC (g/s)
FI	53.2 ± 1.6	286.7 ± 8.4	15.1 ± 1.1	40.3 ± 2.1	10.2 ± 5.3	29.8 ± 14.2
FII	41.3 ± 8.6	393.2 ± 118.5	23.9 ± 3.5	55.2 ± 7.5	21.5 ± 5.6	50.8 ± 11.2
FIII^*∗*^	2887.2 ± 254.3	11376.0 ± 936.0	101.8 ± 3.6	136.7 ± 28.0	77.6 ± 21.2	116.0 ± 19.1

*n* = 3, AUC, area under the curve; FI: film with 10.0% of jambu's extract and 1.0% of macela's essential oil; FII: film with 15.0% of jambu's extract and 1,5% of macela's essential oil; FIII: placebo film (^*∗*^ *p* < 0.05).
